# Freeze-Drying of Blueberries: Effects of Carbon Dioxide (CO_2_) Laser Perforation as Skin Pretreatment to Improve Mass Transfer, Primary Drying Time, and Quality

**DOI:** 10.3390/foods9020211

**Published:** 2020-02-18

**Authors:** Pablo Munzenmayer, Jaime Ulloa, Marlene Pinto, Cristian Ramirez, Pedro Valencia, Ricardo Simpson, Sergio Almonacid

**Affiliations:** 1 Departamento de Ingeniería Química y Ambiental, Universidad Técnica Federico Santa María, 2390123 Valparaíso, Chile; pablo.munzenmayer@alumnos.usm.cl (P.M.); jaime.ulloam@alumnos.usm.cl (J.U.); cristian.ramirez@usm.cl (C.R.); pedro.valencia@usm.cl (P.V.); ricardo.simpson@usm.cl (R.S.); 2 Centro Regional de Estudios en Alimentos y Salud (CREAS) Conicyt-Regional R06I1004, 31000000 Valparaíso, Chile

**Keywords:** blueberry freeze-drying, berry-busting, skin perforation, primary drying time, quality

## Abstract

Freeze-dried berry fruits are generally consumed as they are, whole and without peeling or cutting, as the conservation of their original shape and appearance is often desired for the final product. However, usually, berries are naturally wrapped by an outer skin that imparts a barrier to vapor flow during freeze-drying, causing berry busting. Photo-sequence, experimental, and theoretical methodologies were applied to evaluate the application of CO_2_ laser microperforations to blueberry skin. Under the same set of freeze-drying conditions, blueberries with and without perforations were processed. The results showed that the primary drying time was significantly reduced from 17 ± 0.9 h for nontreated berries to 13 ± 2.0 h when nine microperforations per berry fruit were made. Concomitantly, the quality was also significantly improved, as the percentage of nonbusted blueberries at the end of the process increased from an average of 47% to 86%. From a phenomenological perspective, the analysis of the mass transfer resistance of nontreated fruits, in agreement with reported studies, showed a Type II curvature, with a sharp decrease at low time, followed by a linear increase. In contrast, blueberries with nine perforations depicted a Type III regime, with a saturation curvature toward the time axis. It was demonstrated that CO_2_-laser microperforation has high potential as a skin pretreatment for the freeze-drying of blueberries.

## 1. Introduction

Freeze-drying (FD) or lyophilization is a dehydration method that has been proven to produce high-quality fruit and vegetable products, as compared with other drying processes. Since it is carried out at a low temperature (typically a maximum of 38 to 54 °C shelf temperature), product damage is minimized. Thus, it is used for delicate, high-sensitive, and high-value products to maintain their color, flavor, shape, and nutritional characteristics [1,2,3]. Examples of these kinds of foods are berry fruits such as blueberries, strawberries, maquiberries, and cranberries, among others. Freeze-dried berry fruits are generally consumed as they are, whole and without peeling or cutting, as conservation of their original shape and appearance is often desired for the final product [4]. However, most of these berries are naturally wrapped by a waxy outer skin that imparts barrier to water vapor flow during FD, as happens in all drying processes. This mass transfer limitation is often the phenomenological factor that controls the processing time, energy consumption, and concomitant final quality. In some processes, such as FD, where fruits are exposed to high-energy inputs and/or a high-vacuum environment, a skin rupture or a busting process is frequently observed due to pressure lift just under the skin. As a result, besides the long processing time, many freeze-dried berries, while maintaining most of their original nutritional quality, cannot keep their original shape and appearance, diminishing their organoleptic characteristics significantly [5,6,7,8].

Several skin pretreatment methods have been tested to facilitate water vapor transfer in drying processes, most of them focused on reducing the processing time of Osmotic Dehydration (OD). Physical and chemical methods, such as maceration of the outer skin by knife blade, chemical additives, or needle perforation, have been reported [1,9,10,11,12,13,14]. Due to the presence of undesirable compounds (NaOH, HCl, ethyl oleate), the generation of waste material, and the low quality found in the final product, chemical treatments are not a real alternative to weaken the berry skin. For example, Grabowski and Marcotte [13], reported that chemical skin pretreatment in OD of cranberries resulted in the lowest value of taste acceptability. Different results were reported by Ketata et al. [15]: when liquid nitrogen pretreatment was applied to the OD of blueberries, the dewaxing of the fruit skin allowed a reduction in the drying time of 45% to 65%; however, this was accompanied by a significant loss of total phenolics. Mechanically cutting fruits is another alternative that has been tested in blueberries and tomatoes. However, due to the softness of these fruits, many problems have been observed, including significant damage to the final product, loss of nutritious fluids, and not having the whole fruit as the final product [1]. Alternatively, skin needle perforation has been tested on cranberries and cherry tomatoes. It has been reported that to obtain a significant mass transfer enhancement in OD, 20% to 30% of the total surface area of cranberries should be perforated [13]. Azoubel and Murr [10] perforated cherry tomatoes with 1 mm diameter needles with a pin hole density of 16 holes/cm^2^ prior to OD and air drying, attaining a significant time reduction when more than 80 holes/cm^2^ were used.

Scharschmidt and Kenyon [16] reported the results related to skin pretreatment in FD processing where blueberries were perforated by needles to a density of 2–3 punctures per berry. The main results were that the berries kept their original spherical shape without physical changes to their outer integument, which was declared to be a problem in nonperforated blueberries. In addition, the FD time was two-thirds or less than that of the control process without puncturing. However, no information was given on processing conditions (temperature and pressure), and also the needle diameter was not specified. Thus, even with some time-limited information, physical skin perforation of the whole berry fruit (i.e., blueberry) seems to be a suitable alternative to more efficiently carry out mass transfer processes that reduce the processing time, cost, and energy requirement and avoid product explosion or bust, so that the fruit can retain its basic shape.

Collaterally, puncturing has been tested by utilizing carbon dioxide (CO_2_) laser beam technology to carry out surface microperforations. This has important advantages, and it is already used in many fields such as medicine, cosmetics, and marking industries. Due to its superior accuracy, environmental cleanliness, and safety, applications can be carried out at precise locations, in multiple surfaces and arrangements, and within a size range of 50 to 300 μm [17,18]. Also, it is a noncontact technology that significantly mitigates the chance of physical and microbiological contamination of materials that are typically associated with traditional cutting or contact devices or fluids [19,20]. Fujimaru et al. [9] reported the application of CO_2_ laser microperforation of blueberry skin as a pretreatment in OD. The study demonstrated that CO_2_ laser microperforation can be a viable skin pretreatment which offers notable improvements in water removal; it almost doubled the moisture loss in the first 24 h of OD, from 7% (control) to 11% (2.5 mm square grid perforation), which was even better than the 9% water loss for blueberries that were mechanically cut. This already-proven technology, which the food industry has not completely incorporated, is an attractive alternative that can be applied to berry FD in order to overcome the problems associated with its outer skin.

From a theoretical point of view, the busting process is a consequence of a high initial mass transfer resistance due to the presence of the skin, with this being probably the most significant variable governing water vapor flow during FD and its implications. When the resistance to mass transfer needs to be estimated, the Manometric Temperature Measurement (MTM) methodology has been proven to be an effective tool. Moreover, Pikal et al. [21], concluded that a high resistance to mass transfer at the beginning of the sublimation process is due to the presence of a surface barrier resulting from a structure different from that of the dried layer, a conclusion that has been supported by scanning electron microscopy. This observation may be applied to berry FD, but it needs to be tested.

Thus, the objectives of this study were as follows: (1) to describe the busting process of blueberry freeze-drying through experimental and phenomenological approaches, and (2) to evaluate the use of CO_2_ laser microperforation technology to reduce the processing time as well as product explosion and its associated consequences.

### 1.1. Theoretical Background

#### 1.1.1. The Freeze-Drying Processes

**Figure 1:**
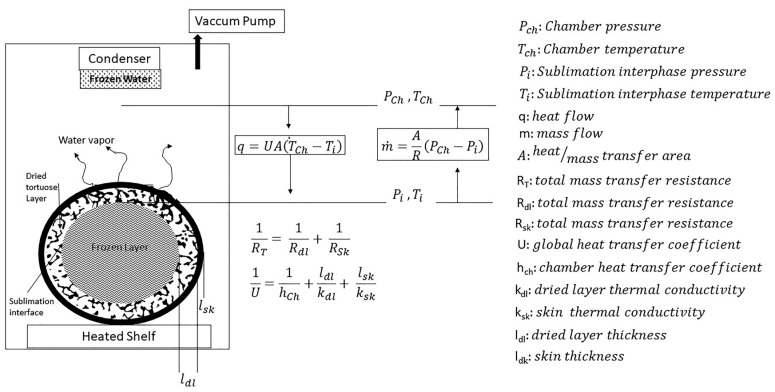
Like-berry-fruit freeze-drying system and its basic phenomenological description.

Similar to other dehydration processes, FD includes simultaneous heat and mass transfer. A simple but conceptual explanation of the phenomenological processes taking place during a typical like-berry-fruit FD operation is described in Figure 1.

A product, initially completely frozen, is placed into a freeze dryer chamber. Then, the chamber pressure is lowered under the water triple point, and heat is supplied by mean of a heated shelf and/or the surrounding air temperature (primary drying or sublimation). As heat flows through the dried layer through heat transfer, sublimation latent heat is delivered, and a sublimation interface recedes leaving, resulting in a thicker porous layer of dried material, which acts as a resistance to heat transfer, as well as water vapor flow, toward the product surface through mass transfer, and finally, to the condenser trap, where water is separated before gases are expelled by the vacuum pump. Both heat and the mass transfer rate are the essential causes that make the FD process slow down. In FD, if heat supplied to the sublimation interface is equal to the latent heat associated with the sublimation rate of ice (m × ΔH_S_ = q), the saturated pressure of sublimation (P_i_) and its corresponding temperature (T_i_) become stable, and the sublimation proceeds normally. However, if the heat supplied to the sublimation interface is not enough, the sublimation rate will drop; conversely, if the total resistance to vapor diffusion is too large (R_T_), then P_i_ and T_i_ may rise. As a result, frozen water will melt and collapse, and/or bust may take place [20,21]. During berry-fruit FD processing, in addition to the dry-layer resistance (R_dl_), the skin operates as an important barrier (R_sk_) that further extends the processing times. Additionally, the low skin permeability can generate explosion or bust problems, as vapor cannot escape at the needed rate so as not to provoke a pressure lift just under the skin [5].

#### 1.1.2. Mass Transfer Resistance

**Figure 2:**
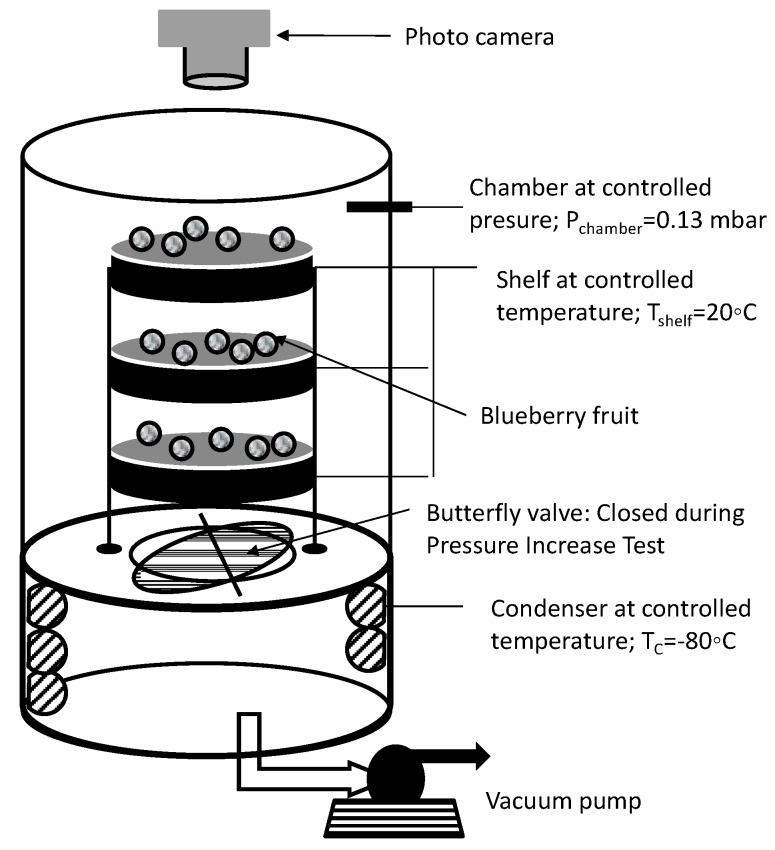
Freeze-drying system arrangement and its processing condition set up.

The estimation of R_T_ through experimental and theoretical approaches would explain and describe the phenomenological process associated with the berry busting process. The interaction effects between the blueberry fruit characteristics, such as mass transfer area (A) the product mass transfer resistance (R_T_), and the freeze-drying processing conditions, have been studied by using the semiempirical MTM procedure, which was originally developed to assess the temperature of the sublimation interface within the product and the dried-layer mass transfer resistance. The method has a large body of literature information [22,23,24,25,26,27]. The principle of MTM is based on the flow of water vapor from the product chamber to the condenser being momentarily interrupted during the primary drying time. The MTM is based on a rapid increase in the chamber pressure by closing the freeze-dryer butterfly valve (Figure 2). During this perturbation process, the chamber pressure will rapidly increase due to the continued sublimation of ice. Since the composition of the vapor phase in the chamber is nearly all water vapor, sublimation will stop when the chamber pressure reaches the vapor pressure of ice at the sublimation interface (diving force ΔP = 0); consequently, the pressure rise will cease. The dynamics of this pressure rise process can be theoretically described by equation (1), which is derived from the phenomenological heat and mass balance over a system, defined as the void volume of the product chamber [28]:

(1) $$P\left(t\right)=P_{i}-\left(P_{i}\;-{\;P}_{0}\right)\mathrm{Exp}\left(\frac{-{\mathrm{NART}}_{s}}{M_{H_{2}0}{\mathrm{VR}}_{T}}t\right)\;+\;0.0465P_{i}\Delta T\left[1-0.811\mathrm{Exp}\left(\frac{-0.114}{L}t\right)\right]+\mathrm{Xt}$$

where P(t) is the system pressure (void volume of the freeze-drying chamber), P_i_ is the interphase pressure of the product, P_0_ is the initial pressure of the system, N is the number of fruit units, M_H2O_ is the water molecular weight; V is the void volume of the freeze drier system, R is the universal gas constant, T_S_ is the shelf temperature, A is the mass transfer area per fruit unit, R_T_ is the total area normalized product resistance to mass transfer, ΔT is the temperature gradient across the frozen layer—normally fixed at 1 K, L’ is the geometric product characteristic—ice thickness, and X is the linear term in equation (1) associated with external air infiltration. Tang et al. [28] generated simulations of pressure rise curves to analyze the relative contribution to the chamber pressure rise (P(t)), of the many terms in Equation (1), concluding that a resistance-dominated period, which takes place in the first 5 to 10 s of the pressure rise, is mainly expressed by the first exponential term, so the others terms are nearly neglected as part of the summation.

## 2. Materials and Methods

### 2.1. Materials

The experiments were carried out with fresh blueberries of the Huertos de Chile brand (variety *Vaccinium corymbosum*), which were bought at a local market. Blueberries with a diameter of 14–15 mm were selected.

### 2.2. Blueberry Characterization and Freezing

The blueberry fruits used as raw material to carry out a set of experimental treatments were characterized to ensure a homogeneous quality of said material. Total Soluble Solids (TSS), or °Brix, was selected for this purpose as it is one of the main quality indicators of the blueberry fruit [29]. To determine °Brix, blueberry fruits were weighed using a RADWAG balance (AS 220/C/2, Radom, Poland) and then crushed and homogenized for 5 min with 10 mL of distilled water until a homogeneous juice was obtained. The resulting juice was filtered, and aliquots were taken for °Brix analysis with a HI 96,680 digital refractometer (Hanna instruments, Rothe Island, USA).

Blueberries to be used in the set of experimental treatments were first frozen in a convection freezer (MT05, Alaska, Minas Gerias city, Brasil) at −40 °C for 2 h to obtain a product temperature equal to or less than −35 °C.

A series of experiments, divided into two steps, was carried out in order to achieve the research objectives:

(i) (Blueberries were freeze-dried without any treatment, where the busting process was observed through photographic monitoring. The fraction of busted blueberries was computed throughout time.
(ii) The same freeze-drying process was applied to blueberries under different treatments:
  - Whole, without any treatment.
  - Cut in half.
  - With 1, 3, 6, and 9 CO_2_-laser microperforations.

The Pressure Increase Test (PIT) was performed to estimate the treatment effect on primary drying time, and the MTM method was applied to evaluate the mass transfer phenomena, as affected by blueberry characteristics (skin, A, R_T_) and the busting process. The final fraction of busted blueberries was also evaluated. Table 1 depicts a summary of the experimental setup, showing its two steps—visual observation/evaluation of the busting process of whole blueberries and a total of 6 treatments to evaluate the effect of CO_2_-laser microperforations on primary drying time and quality.

### 2.3. Freeze-Drying Process

All experiments were carried out in a Martin Christ freeze-dryer, model Alpha 2-4 LSCplus (Martin Christ Gefriertrocknungsanlagen, Osterode, Germany), which can operate at a total vacuum pressure of up to 0.001 mbar, provided with an MKS Baratron 622 capacitance manometer (MKS Instruments) and a condenser that can operate at temperatures down to −85 °C (see Figure 2). It has three shelves of 0.021 m^2^ each that can be temperature controlled with a wireless temperature monitoring system, which, in turn, allows for sample temperature monitoring through pt100 port sensors. The samples or blueberries were placed onto the three shelves, considering a total load of 60 units. In order to assess the product temperature, one blueberry fruit per shelf was inserted with a miniature pt100 sensor (PT 100 Mini-Epsilon LSC Plus, Martin Chris, ostero de am Harz, Germany). A standard freeze-drying processing condition was fixed at 0.13 mbar (13 Pa) with a condenser temperature of −85 °C and a shelf temperature of 20 °C, while the air temperature was also 20 °C. The freeze-drier system includes dedicated software (SCADA Software V1 LPC plus Martin Chris, ostero de am Harz, Germany) to set, control, and monitor the freeze-drying process at the selected processing condition. It samples data every 5 s. A butterfly valve with an approximate closing time of 0.5 s, mounted in the cylindrical duct connecting the freeze-drying chamber and the condenser chamber, can be managed by the software to perform Pressure Increase Tests (PIT) (Figure 2). The SCADA Software LPC plus software allows graphic visualization and recording of several process variables: tray temperature, blueberry temperature, condenser temperature, chamber absolute pressure (capacitive sensor), and PIT data.

### 2.4. Determination of Primary Drying Time

After freezing, the FD process proceeds to primary-drying or sublimation, and finally to secondary drying or water desorption. The primary drying stage—conventionally, the most time-consuming part of the process—was investigated in the present study. In order to determine the end of the primary drying time or the transition from the primary drying stage to the final secondary-drying, which can be estimated with the automatic Pressure Increase Test (PIT), the freeze-drier software system was implemented. It works by temporarily closing the butterfly valve between the product chamber and the ice condenser (see Figure 2), allowing a pressure increase in the product chamber. If the pressure increase remains below a set limit, usually 10% whilst the valve is closed, the software program assumes that there is no further sublimation water left in the product, and the primary drying phase can be considered to be finished, and this time is recorded. Another way to estimate the end of the primary drying stage is to observe the difference between the shelf and assessed product temperature; when they equalize, no more sublimation heat is absorbed, meaning the sublimation is over.

### 2.5. Visual Registry of the Blueberry FD Process

A photo-sequence methodology was used to capture and visually analyze the dynamics of the busting process while blueberries are being freeze-dried. Images of the top-shelf blueberries were taken by a photographic camera (Flea^®^3 FL3-GE-03S2C-C Color GigE Camera, FLIR Systems, Wilson Ville, OR, USA) located at the upper part of the freeze drier system (Figure 2). A dataset of images taken at a rate of 12 photographs per min was then visually inspected to calculate the fraction of busted blueberries over time.

### 2.6. Estimation of the Dry-Layer Mass Transfer Resistance (R_T_)

The MTM method was used to evaluate the influence of product characteristics in the FD process. The needed chamber pressure-rise as a function of time can be experimentally monitored, and the acquired data can then be regressed through Equation (1), where unknown parameters such as P_i_, X, A_T_ (where A_T_ = N × A_unitary_), and R_T_ can be estimated. The minimum conditions on the experimental procedure have been reported in order to obtain reliable results. The data collection time could not be longer than 30 s, since a longer time would allow a considerable product temperature increase because of the closed system, and within that time, there needed to be sufficient data collection to observe the complete development of the exponential part of Equation (1), which accounts for the pressure rise controlled by the product resistance. During pressure increase runs, the dynamic pressure increase was monitored with a cDAQ module/9215 data acquisition system and LabVIEW software, manufactured by National Instruments (11500 N Mopac Expwy, Austin, Texas, United States), allowing a sampling period of as low as 10 ms. To successfully apply the MTM method, the product of the geometric characteristics and the particular void volume of the chamber system needed to be adjusted. It has been demonstrated that the computed value of the exponential expression Q (without considering t) should be equal to or higher than 0.2 (1/h) to ensure complete depiction of the exponential phase of the pressure rise in the product chamber [28]:

(2) $$Q=\left(\frac{3.461{\mathrm{NAT}}_{s}}{{\mathrm{VR}}_{T}}\right)\geq 0.2\;(1/h)$$

For a given value of R_T_, Q allows the minimum number of units (N, number of blueberry fruits) that must be loaded in a particular freeze-drier system (characterized by its void volume V) to be estimated to obtain a value equal to or higher than 0.2 (1/h). A total resistance value (R_T_) of approximately 3 (torr h cm^2^/g) has been reported to be acceptable to carry out this assessment. 

### 2.7. CO_2_ Laser Microperforations and Laser System Settings

As mentioned in the introduction section, CO_2_ laser microperforation is a convenient pretreatment as it is a noncontact technology that significantly mitigates the chance of physical and microbiological contamination of materials that are typically associated with traditional cutting or contact devices or fluids. In this respect, the most-used pretreatment technology is to machine-cut the blueberry fruits into halves, which effectively avoids the busting process and reduces the primary drying time. Then, both pretreatment technologies were evaluated: CO_2_ laser perforation at four levels of perforation—1, 3, 6, and 9 perforations per blueberry fruit—and FD blueberry cut into halves. Finally, the results were compared with each other and with those of FD whole blueberries.

A 100 W CO2-laser system (Firestar t100, Synrad Inc., Mukilteo, Wash., U.S.A.) was used to carry out the perforations of blueberries. The system was equipped with a 125 mm focusing lens (FH series Flyer, Synrad Inc.) and a computer interface with laser marking software (WinMark Pro, Synrad Inc.).

The system was operated at a continuous wavelength of 10.6 μm and a frequency of 10 kHz. Perforations were made in a square grid pattern with a density of 2.0 × 2.0 mm.

The CO2 laser was set at 120 pulses, duration of 1 millisecond, linear speed of 100 cm per second, distance of 128 mm between the laser and the surface of the blueberry, and a percentage of power that was determined experimentally by observing in a microscope (Helmut Hund GmbH, H600/12) the depth of the perforation, until finding the configuration of the laser that allowed us to perforate until 1/3, varying the power of the laser (whose maximum was 100 watts).

A unit of frozen (−35 °C) blueberry was loaded onto an aluminum tray (20 cm × 20 cm) to perform microperforations; then, blueberry samples were refrozen at −35 °C and kept until FD experiments. 

## 3. Results and Discussion

### 3.1. Blueberry Characteristics

Blueberry size was selected within the diameter range of 14 to 15 mm. Assuming a spherical shape for the blueberry fruit, and, given a diameter of 14.5 mm (1.45 cm), its surface area ($A=4\pi r^{2}$) results in a unit area of 6.6 cm^2^.

Total Soluble Solids of the blueberries used as raw material for experimental procedures showed a characteristic quality indicator within the narrow enough range of 9.4 ± 0.7 °Brix.

### 3.2. Busting Process of Nontreated Whole Blueberries

The whole blueberry FD process was set as described in the methodology section.

**Figure 3:**
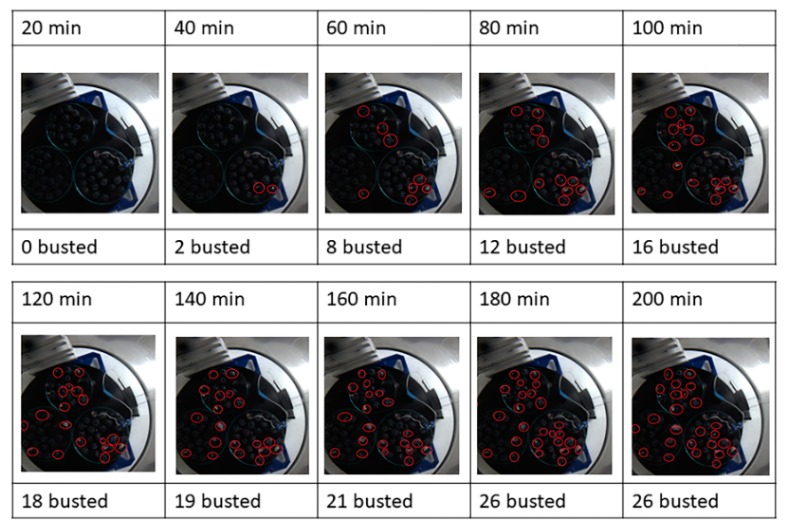
Visual evaluation of the busting process. Inspection of photographs taken at different times during the freeze-drying of whole nontreated blueberries.

A slide dataset that consists of images extracted from the photo sequence every 20 min was used to visually quantify the fraction of busted blueberries over time; this evolution is depicted in Figure 3. The busted blueberries are identified by red circles, reflecting the progression of the busting process, which starts with zero busted at 20 min and describes a saturation curve, achieving a maximum of 26 busted blueberries at 180 min. 

**Figure 4:**
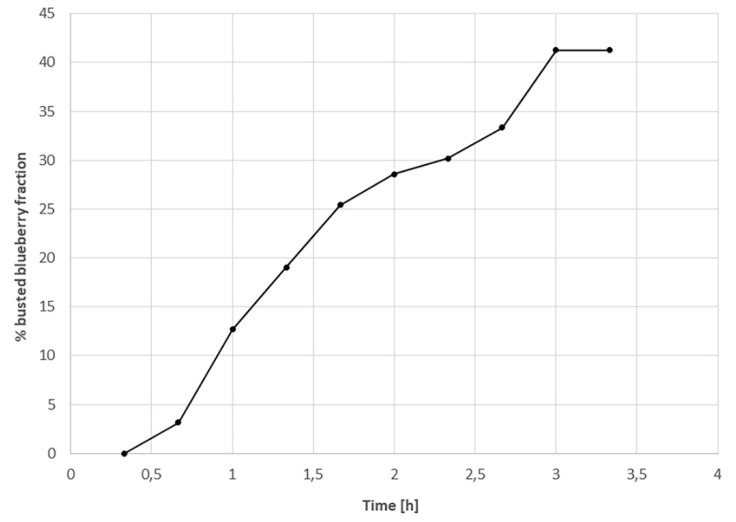
Busting process dynamics as evaluated from photographs taken during the freeze-drying process of whole nontreated blueberries.

**Figure 5:**
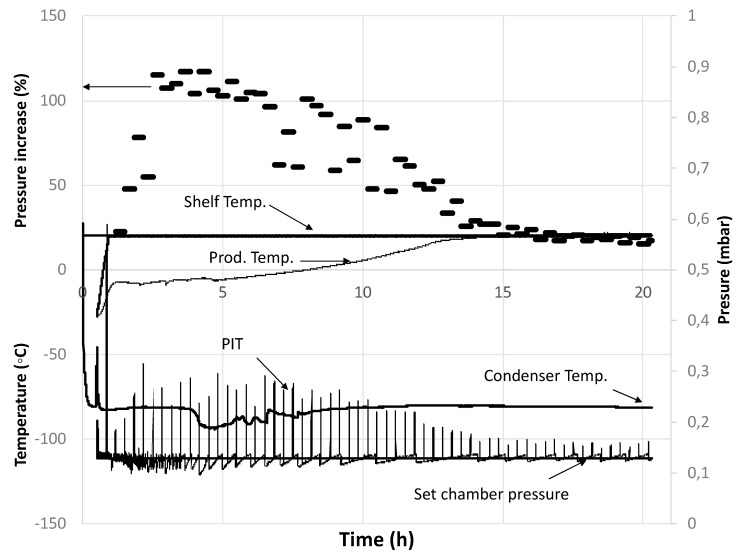
Main processing variables and measurements during the freeze-drying of whole nontreated blueberries, PIT.

Note that the dynamic of the busted blueberry percentage increased by up to 43% after 3 h of freeze-drying, and then it stayed constant (Figure 4). This means that the resistance to the mass flow of sublimated water is high at the beginning, and then it decreases as the number of busted blueberries increases. Then, a normal FD process should continue with increasing resistance as the dried layer gets thicker. The main process parameters and measurements of the whole blueberries FD, treatment 1 in Table 1, are depicted in Figure 5.

As expected, the percentage pressure increase (PIT) was low at the beginning of the process until around 2.5 h, in agreement with that shown in Figure 4. Then, it stayed approximately constant for 2.5 h, before decreasing at a moderate rate until 13 h, and finally, a very low decrease continued to get down to values under 10% after around 17 h. The product temperature also achieved the shelf temperature at approximately 15 h. Two important outcomes can be extracted from this result. First, normally, the resistance to vapor flow from the FD product is low at the beginning of the process, as no dried layer exists; however, in this case, resistance seems to have been high at the process initiation due to the high skin resistance. Then, as the busting process proceeds, the resistance goes down, before finally increasing again as the dried layer develops. Secondly, the results indicate that the primary drying time is in the range of 15 to 20 h. From the experiments carried out in triplicate, the end of the primary drying time was assessed by the product of the thermocouple response and PIT, and there was good agreement between the two techniques, with the global primary drying time being 17 ± 0.9 h with a final moisture content of 14% ± 2%.

In order to quantify the effect of the blueberry skin on mass transfer, the MTM method was applied. It has been demonstrated that the computed value of the exponential expression of Equation (1) (Q; without considering t) should be equal to, or higher than, 0.2 (1/h) (Equation(2)) to ensure complete depiction of the exponential phase of the pressure rise in the product chamber. This phase reflects the influence of the resistance (R_T_) on the mass transfer phenomena. For a given value of R_T_, Equation (2) allows us to estimate the minimum number of fruit units N (number of blueberry fruits) that must be loaded in a particular freeze-drier system (characterized by its void volume V). A value of R_T_ of approximately 3 (torr h cm^2^/g) has been reported to be acceptable to carry out this assessment [28]. To satisfy the above condition, the following set of values were considered:

R/M_H2O_ = 3.461 (torr L/g-K);

N ≥ 60 units;

A = 6.6 (cm^2^/unit);

V = 32.5 (L);

T_S_ = 293 K;

R_T_ = 3.0 (torr h cm^2^/g).

These data were input into Equation (2), resulting in a Q value of 4.1 > 0.2.

**Figure 6:**
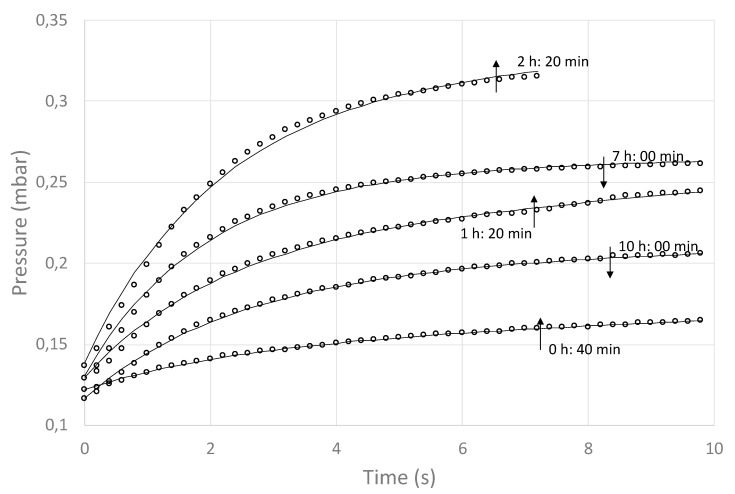
Fit of MTM equation to experimental pressure rise profiles for progressive times along the freeze-drying process of whole nontreated blueberries.

All published studies about the application of the MTM method have used a cylindrical shape (vials in the pharmaceutical industry), where the mass transfer area (A) does not change as the FD proceeds (one-dimensional axial mass transfer). However, assuming a spherical geometry to represent the blueberry shape, area A does change over time. For this reason, in this study, the quotient (A_T_/R_T_), was taken as a regression variable, together with P_i_ and X (see Equation (1)). Experimental data of the pressure rise vs. time over 30 s were generated every 20 min. The pressure rise data were taken at a rate of 100 data/s and then averaged to generate a data set of 5 data points per second. These data were then regressed against Equation (1) to obtain (A_T_/R_T_). In order to show the evolution of the process, Figure 6 depicts the first 10 s of some of the pressure rise vs. time experimental data and their respective regression curves. As expected, the regressions showed neglected values for variable X (in the range of 0.001 to 0.0001), indicating a good seaming of the FD system (no air infiltration). Also, the change of P_i_ values was minor in comparison with the changes of (R_T_/A_T_), meaning that the latter is the main agent of chamber pressure rise [28]. 

**Figure 7:**
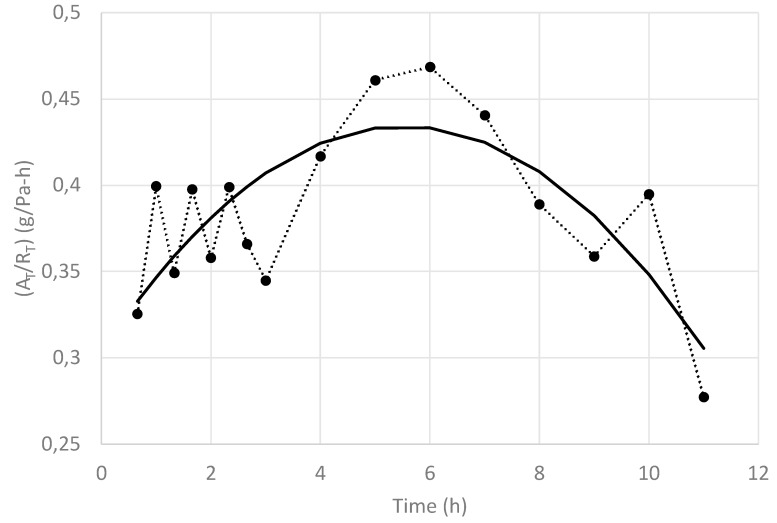
Values of (A_T_/R_T_) along the freeze-drying time (•) and its corresponding tendency line (**—**) of whole nontreated blueberries.

It can be seen that at the beginning (0 h 40 min), the pressure rise was low, and it kept increasing up to 2 h 20 min. Then, it started to decrease, approximating the pressure rise observed at the process initiation (10 h 00 min). The (A_T_/R_T_) values obtained by the regression analysis at different processing times, together with a tendency line, are shown in Figure 7.

**Figure 8:**
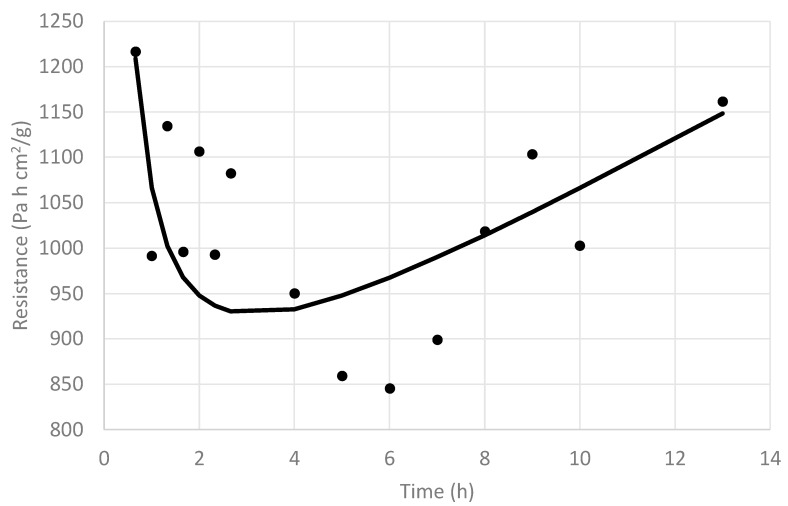
Values of (R_T_) along the freeze-drying time (•) and its corresponding tendency line (**—**) of whole nontreated blueberries.

This value started at a low value, which means that a high R_T_ value has an important effect in the quotient (A_T_/R_T_), and it increased rapidly over time up to 4 h, as some of the blueberry fruits busted and R_T_ decreased, in spite of the A_T_ value that should have been decreasing during this period. This reveals that the main effect is that given by the important reduction of R_T_ (busting process). If an average A_T_ over time is assumed (for example, A_T_ = 31.4 cm^2^), the resistance R_T_ as a function of time can be approximated, and its result is shown in Figure 8, including a tendency line, where, contrary to a normal FD process, R_T_ started at a high value and then decreased sharply before finally increasing again.

This observation reflects the presence of a surface barrier, which appears to be the blueberry skin, which, after being exposed to increasing vapor pressure for some time, cracks, and the effective resistance decreases. This event does not occur at the same time for all FD blueberry fruits, resulting in the sharp, but not instantaneous, R_T_ reduction. This is in agreement with what was observed by Lu and Pikal [30], Pikal et al. [21], and Pikal [23], where some experiments using water solutions with different solutes showed initial values of R_T_ significantly larger than zero, which suggests a surface barrier resulting from a different structure for the dried product near the surface, which was attributed to the formation of a high-resistance “skin” during the freezing step of the FD process, a conclusion that was supported by scanning electron microscopy. These authors, after performing various experiments with different compositions, suggested four types of resistance curves: Type I, with a linear dependence of R_T_ on the dried layer thickness (or concomitant elapsed time); Type II. with a sharp decrease in R_T_ after a short period of time, followed by a linear increase in R_T_ with increasing time; and Type III and IV, with a saturation curvature toward the time axis, with Type III being less severe than Type IV. The bust process described in this study is clearly a Type II resistance curve.

These results indicate that the reduction of the initial value of R_T_, considering a pretreatment to the blueberry skin, would enhance the water vapor flow at the beginning of the process, and consequently would reduce the primary drying time and/or increase the quality yield by bringing down the fraction of busted blueberry units at the end of the process.

### 3.3. Evaluation of CO_2_ Laser Microperforation and Blueberries Cut in Half

**Figure 9:**
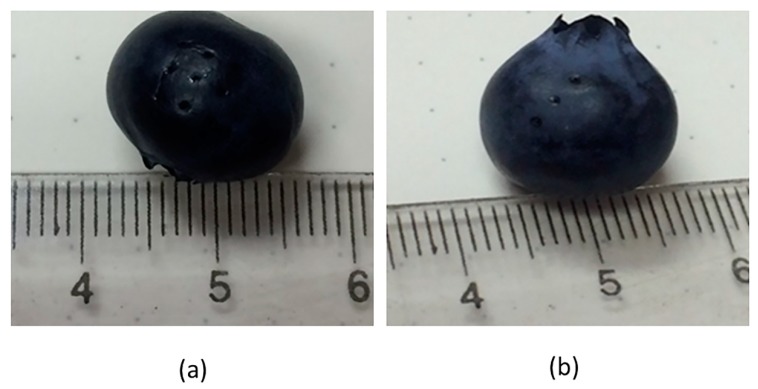
Appearance of blueberry fruit with nine (**a**) and three (**b**) CO_2_ laser microperforations.

Experimental results were obtained for all five pretreated blueberries: 1, 3, 6, and 9 perforations and cut in half, treatments 1 to 6 in Table 1. Microperforations were made as described in the methodology, obtaining perforations in a square grid of 2.0 mm × 2.0 mm density, 0.5 mm diameter, and 1/3 of the blueberry diameter in depth, by utilizing 19% power (19 W). The FD system was set at the same conditions used for whole nontreated blueberries, and the same number of blueberry units were loaded in each experiment. The final moisture at the end of primary drying was also controlled and was within the range of 13% (95% CI (10%, 17%)) to 15% (95% CI (12%, 18%)), for all treatments. Figure 9 shows a blueberry with nine and three CO_2_ laser microperforations in a square arrangement with 2.5 mm spacing. It demonstrates that the CO_2_ laser microperforation process is minimally invasive with a negligible impact on quality characteristics.

**Figure 10:**
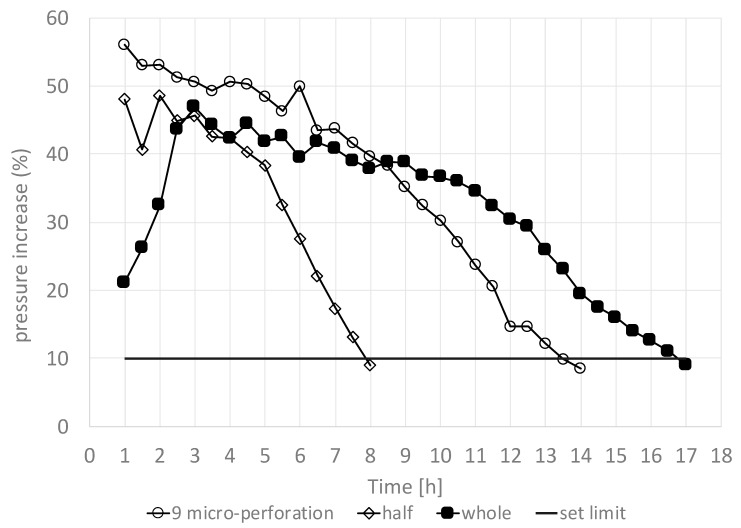
Pressure Increase Test (PIT) applied to blueberry freeze-drying to estimate primary drying time as affected by CO_2_ laser microperforations and cut-in-half treatments.

The PIT and product temperature response were used to evaluate the effect of pretreatment on the primary drying time. Each test was conducted in triplicate to determine the average and standard deviation of the primary drying time. Figure 10 depicts the PIT for the blueberries that were whole and nontreated, cut in half, and microperforated nine times. It is interesting to note that for both cut-in-half and nine-times-perforated blueberries, the initial PIT value started higher than that of the whole nontreated fruit and decreased over time. This is due to the weakened pretreated skin or the area without skin being available to sublimation.

If the pressure increase was equal to or less than 10%, then the primary drying time was considered to be over.

Table 2 shows the results of the primary drying time for the most significant treatments (treatments 1, 6, and 2 in Table 1).

**Figure 11:**
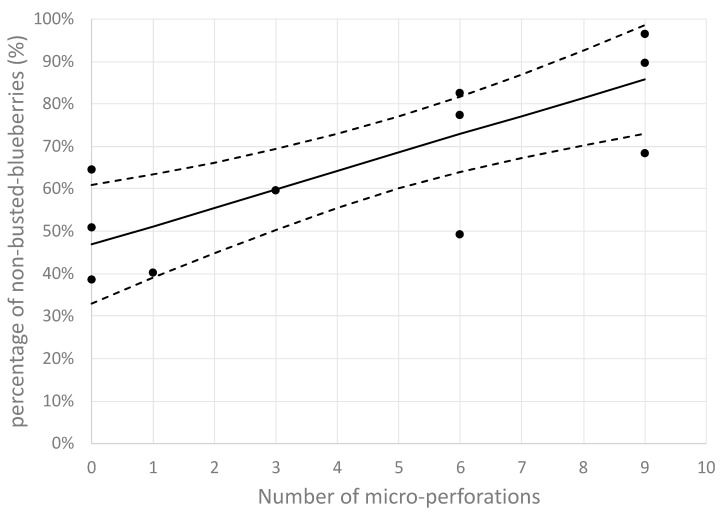
Experimental and statistical analysis of the nonbusted percentage (%) as affected by CO_2_ laser microperforations.

It can be seen that there was an important primary drying time reduction of 60.6% when blueberries were cut in half, as compared with that of whole nontreated blueberries. However, this option is associated with the loss of the important quality characteristic of maintaining the fruits’ original shape. On the other hand, and not less importantly, it can be observed that primary drying time reduced by approximately 23.5% when the blueberry skin was pretreated with nine microperforations, as compared with that of whole nontreated blueberries. This result is explained by the fact that the nine-microperforation pretreatment avoids the initial high resistance (R_T_) at the beginning of the process, mitigating the busting process. This means that, in addition to primary time reduction, CO_2_ laser microperforation pretreatment can improve the final product quality. It can be seen from Figure 11 that the percentage of nonbusted blueberries significantly increased as the number of perforations per fruit increased, which is statistically sustained by a positive value of the covariance (covariance = +0.634). In addition, the average value of the percentage ofnonbusted blueberries with nine microperforations was 86%, significantly higher than 47% for those with no microperforations. This is supported by their corresponding 95% CIs: 

Zero microperforations: average 47%; 95% CI (33%, 61%);

Nine microperforations: average 86%; 95% CI (73%, 99%). 

**Figure 12:**
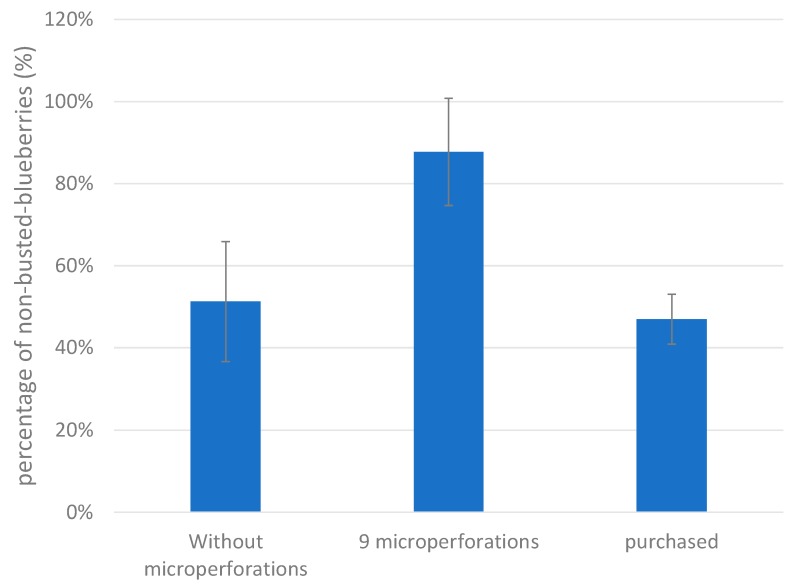
Differences between freeze-dried blueberries as affected by pretreatment and comparison with those that can be found in local markets.

Figure 12 also shows that pretreated blueberries are significantly better than those that can be purchased in a local market.

**Figure 13:**
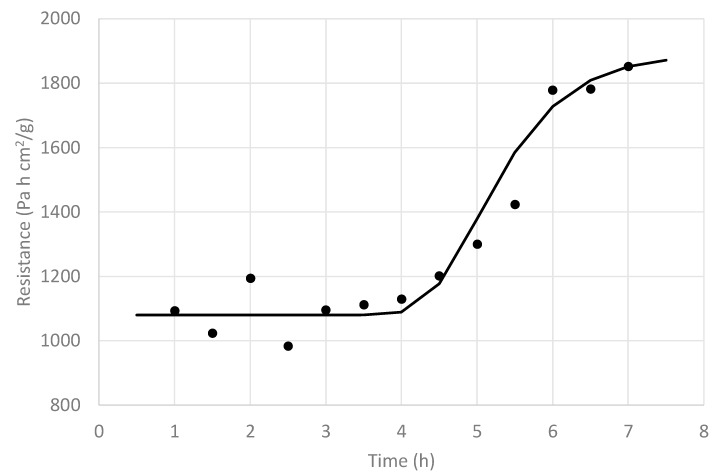
Values of (R_T_) along the freeze-drying time (•) and its corresponding tendency line (**—**) of cut-in-half treated blueberries.

**Figure 14:**
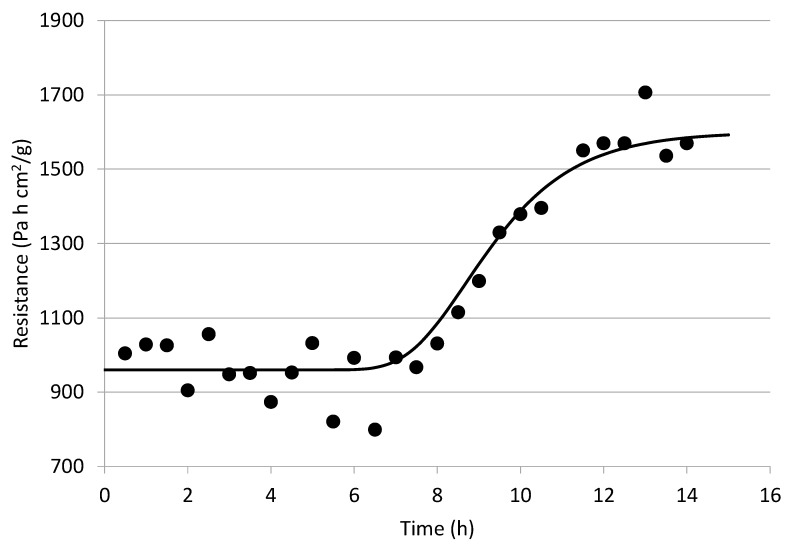
Values of (R_T_) along the freeze-drying time (•) and its corresponding tendency line (**—**) of nine CO_2_ laser microperforations treated blueberries.

Similar to the analysis made to the whole blueberry process, pressure rise data were regressed against Equation (1) to obtain values of (A_T_/R_T_) to finally assess the effect of two pretreatments (nine perforations and cut in half) on R_T_ behavior during the FD process and compare them with that of the whole nontreated blueberry process. Figure 13 and Figure 14 depict the resistance behavior of cut-in-half and nine-perforation treatments over time, respectively. In both cases, it can be observed that there is a sort of lag period for the R_T_ value, which can be attributed to the fact that the skin resistance (R_sk_) is still significantly higher than that of the dried layer, with this lag period being longer for the nine-perforation pretreatment than that of the cut-in-half treatment (about 3 h difference). After the lag period, both of them behave like the normal FD process. 

**Figure 15:**
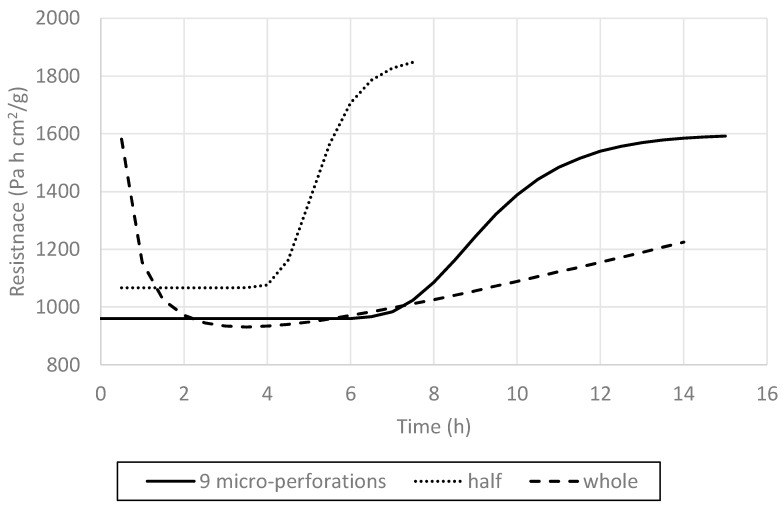
Comparison of mass-transfer resistance profiles as affected by the main treatments.

In order to compare all three treatments—whole nontreated, nine-perforation, and cut-in-half blueberries—the tendency lines were represented in the same graph (Figure 15). This figure summarizes the phenomenological effect of the skin and pretreatments on the blueberry freeze-drying, an effect that is reflected by the R_T_ behavior during FD processing. According to the classification made by Pikal et al. [21], the whole nontreated blueberry FD process, as mentioned, would follow a Type II resistance curve; the cut-in-half blueberries would follow a Type IV process with a severe curvature toward the time axis; and finally, the nine-perforation blueberries would exhibit Type III behavior, with a less severe saturation curvature toward the time axis than that of Type IV. These outcomes phenomenologically describe the effect of the skin and pretreatments on the mass transfer process, which is mainly reflected by the evolution of R_T_ throughout the FD processing time.

## 4. Conclusions

The outcomes from the present study demonstrate that CO_2_ laser microperforation has high potential to be applied as a skin pretreatment for the freeze-drying of blueberries, which offers significant improvement on the process efficiency and final product quality.

The CO_2_ laser treatment allowed the arrangement of microholes that minimally affect the quality and appearance of the fruit, serving as pathways for the flow of water vapor from the sublimating interface through the weakened mass transfer resistance of the berry skin, alleviating the pressure development under it, eventually avoiding the fruit bust, and enhancing the final product quality with a reduced processing time. This makes this novel technological approach an attractive alternative over traditional techniques.

Further research will be required to study other perforation arrangements and perforation depths in blueberries as well as other berry fruits during freeze-drying. The impact on energy consumption and cost-effectiveness should also be evaluated.

## Figures and Tables

**Table 1:** Description of the experimental setup.

	Busting Process Study	Treatment Number					
		1	2	3	4	5	6
Process/Activity							
Blueberry Characterization	•	•	•	•	•	•	•
Whole, no perforation	•	•					
Cut into half			•				
CO_2_-laser 1 perforation				•			
CO_2_-laser 3 perforation					•		
CO_2_-laser 6 perforation						•	
CO_2_-laser 9 perforation							•
Freeze-drying process: 20 °C; 0,13 mbar	•	•	•	•	•	•	•
Photo camera monitoring for visual inspection	•						
PIT test, for Primary-drying time estimation		•	•	•	•	•	•
MTM test, for skin/dry-layer Resistance estimation		•	•				•
Evaluation of busted blueberry fraction	•	•	•	•	•	•	•

**Table 2:** Primary drying time of blueberry freeze-drying as affected by CO_2_ laser microperforations and cut-in-half treatments.

Microperforations	Time [hours]
Without microperforations	17.0 ± 0.9
Nine microperforations	13.0 ± 2.0
Cut in half	6.7 ± 1.2
